# Acute Administration of Desformylflustrabromine Relieves Chemically Induced Pain in CD-1 Mice

**DOI:** 10.3390/molecules24050944

**Published:** 2019-03-07

**Authors:** Loni A. Weggel, Anshul A. Pandya

**Affiliations:** Department of Biosciences, College of Rural and Community Development, 101D Harper Building, 810 Draanjik Drive, University of Alaska Fairbanks, Fairbanks, AK 99709-3419, USA; laweggel@alaska.edu

**Keywords:** desformylflustrabromine (dFBr), nicotinic acetylcholine receptors (nAChRs), positive allosteric modulators (PAMs), pain

## Abstract

Neuronal nicotinic acetylcholine receptors are cell membrane-bound ion channels that are widely distributed in the central nervous system. The α4β2 subtype of neuronal nicotinic acetylcholine receptor plays an important role in modulating the signaling pathways for pain. Previous studies have shown that agonists, partial agonists, and positive allosteric modulators for the α4β2 receptors are effective in relieving pain. Desformylflustrabromine is a compound that acts as an allosteric modulator of α4β2 receptors. The aim of this study was to assess the effects of desformylflustrabromine on chemically induced pain. For this purpose, the formalin-induced pain test and the acetic acid-induced writhing response test were carried out in CD-1 mice. Both tests represent chemical assays for nociception. The results show that desformylflustrabromine is effective in producing an analgesic effect in both tests used for assessing nociception. These results suggest that desformylflustrabromine has the potential to become a clinically used drug for pain relief.

## 1. Introduction

Pain is an unpleasant sensation that can influence the physical as well as the mental wellbeing of an individual [1,2]. Currently, non-steroidal anti-inflammatory drugs (NSAIDs) and opioid analgesics are the main stay for pain relief in clinical settings; however, both drug classes have some drawbacks associated with their use [3,4]. While the chronic use of NSAIDs increases the likelihood of injury to the gastrointestinal tract mucosa, opioid analgesics have a very high addictive liability [5,6]. Newer pharmacological agents with increased analgesic efficacy and relatively fewer side effects are therefore needed for pain relief. One potential target for developing clinically useful drugs for pain relief are the nicotinic acetylcholine receptors (nAChRs) [7,8].

**Figure 1:**
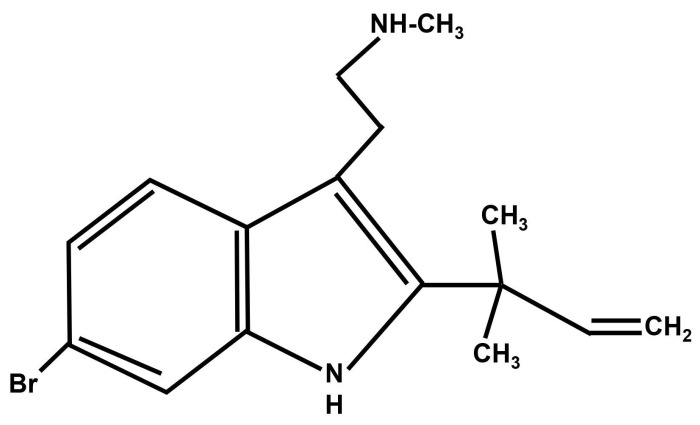
Structure of desformylflustrabromine (dFBr).

The α4β2 subtype of nAChRs are located in the brain and the spinal cord where they are involved in modulating neuropathic and inflammatory pain [9]. In the brain, α4β2 receptors are widely distributed in regions that are associated with the descending monoaminergic inhibitory pain pathway [10]. In addition to the brain, presynaptic α4β2 receptors that are located in the spinal cord inhibit nociceptive neuro-transmission leading to a reduction in the sensation of pain [11]. In a study that used α4- and β2-knockout mice, a decrease in the anti-nociceptive effect of nicotine was observed. There are several other studies that have reported that agonists as well as partial agonists of the α4β2 receptors produce an analgesic effect when assessed in animal models for pain [12,13,14,15,16]. In addition to agonists and partial agonists, positive allosteric modulators (PAMs) of the α4β2 receptors have also been investigated for their ability to induce anti-nociceptive effects in rodents. PAMs are ligands that bind to nAChRs on allosteric sites and potentiate the effects of agonists, but they cannot activate the receptors on their own [17,18]. An increase in the analgesic activity of agonists is observed in rats when they are administered with PAMs of the α4β2 receptors [19,20]. Desformylflustrabromine (dFBr) (Figure 1) is a secondary metabolite obtained from the marine bryozoan *Flustra foliacea* that acts as a PAM for the α4β2 subtype of nAChRs [21,22].

The half-maximal potentiating concentration of dFBr for the α4β2 receptors is around 120 nM [23]. A prior study with dFBr has found that it cannot reduce pain on its own [24]. However, in combination with nicotine, dFBr was able to relieve neuropathic pain in a dose-dependent manner [24].

Chronic exposure as well as acute systemic administration of nicotine cause an increase in locomotor activity in mice, while at the same time such an effect is absent in nAChR β2-subunit knockout mice [25,26]. This fact indicates that the increase in locomotor activity is due to the interactions of nicotine with the β2-subunit containing nAChRs. Since dFBr is a PAM for only those nAChRs that contain the β2-subunit and the principal face of the β2-subunit is involved in dFBr-induced receptor modulation [23,27], in this study, we have investigated the effects of dFBr on locomotor activity as well as on anxiety in the open field exploration test.

The primary goal of this study was to determine if dFBr is able to produce an analgesic effect in chemical assays for nociception in rodents. For this purpose, we utilized the formalin-induced pain test and the acetic acid-induced writhing response test, both of which are chemical assays for studying nociception in rodents.

## 2. Results

### 2.1. Formalin-Induced Pain Test

**Figure 2:**
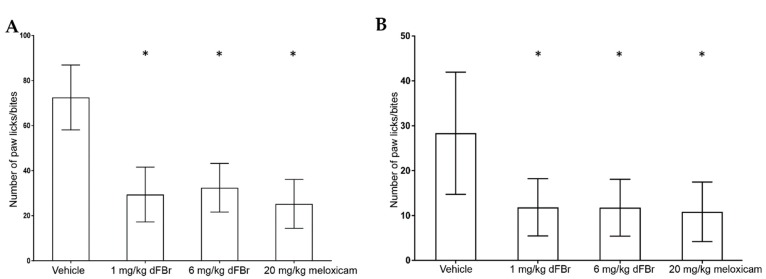
(**A**) Desformylflustrabromine (dFBr) significantly decreases the paw licks/bites in the early phase of the formalin test. The paw licks/bites are expressed as mean (±S.D.). Statistical significance was considered as * *p* < 0.05. Twelve mice were included in each treatment group (*n* = 12). (**B**) dFBr significantly decreases the paw licks/bites in the late phase of the formalin test. The paw licks/bites are expressed as mean (±S.D.). Statistical significance was considered as * *p* < 0.05. Twelve mice were included in each treatment group (*n* = 12).

The formalin test was carried out in two phases: the early phase (Figure 2A) and the late phase (Figure 2B). In both phases, the number of paw licks/bites was used to measure the response to pain. In the early-phase response of the formalin test, both tested doses of dFBr (1 mg/kg and 6 mg/kg) caused a significant reduction in the number of paw licks/bites compared to the vehicle-treated group of mice (F (3.44) = 39.05; *p* < 0.0001). Similarly, in the late-phase response, both 1 mg/kg and 6 mg/kg of dFBr produced a significant decrease in the number paw licks/bites in comparison to the vehicle-treated group of mice (F (3.44) = 11.06; *p* < 0.0001).

A dose of 20 mg/kg of meloxicam was used as a positive control for pain in this study. The number of paw licks/bites observed with 1 mg/kg and 6 mg/kg of dFBr was similar to that observed in mice treated with 20 mg/kg meloxicam in the early (F (2.33) = 0.7993; *p* = 0.4581) and late phases (F (2.33) = 0.089; *p* = 0.9150) of the test. In both phases of the formalin test, the number of paw licks/bites seen with 1 mg/kg and 6 mg/kg of dFBr was similar, with no significant differences among them (early phase (*p* = 0.5299); late phase (*p* = 0.9747)).

### 2.2. Acetic Acid-Induced Writhing Response Test

**Figure 3:**
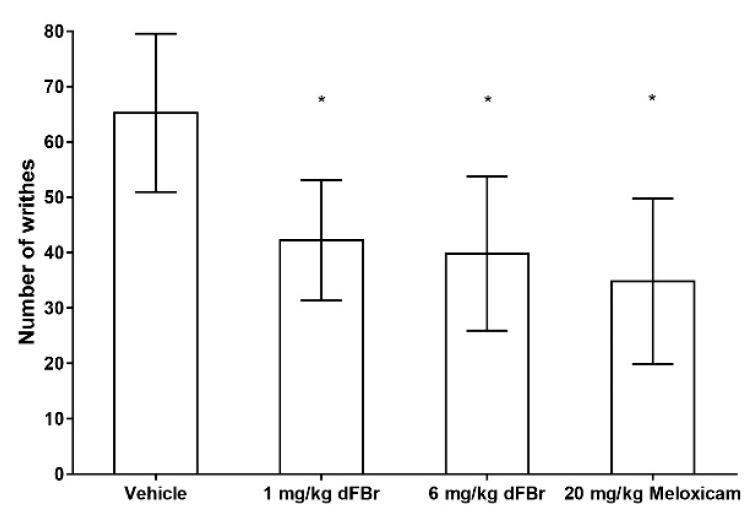
Desformylflustrabromine significantly decreases the number of writhes in the writhing test. The number of writhes is expressed as mean (±S.D.). Statistical significance was considered as * *p* < 0.05. Twelve mice were included in each treatment group (*n* = 12).

In the writhing response test, the number of writhes were used as an indicator of the pain response in the mice. The results of the writhing test were similar to those obtained with the formalin test. Both 1 mg/kg and 6 mg/kg of dFBr were able to bring about a significant decrease in the number of writhes in comparison to the vehicle-treated group of mice (F (3.44) = 11.80; *p* < 0.0001) (Figure 3). The number of writhes seen with 1 mg/kg and 6 mg/kg of dFBr was also similar, with no significant differences among them (*p* = 0.6405). Similarly, the number of writhes observed with 1 mg/kg and 6 mg/kg of dFBr was similar to that observed in mice treated with 20 mg/kg of meloxicam (F (2.33) = 0.9604; *p* = 0.3932). However, the least mean number of writhes was seen in the meloxicam-treated group of mice.

### 2.3. Open Field Exploration Test

**Figure 4:**
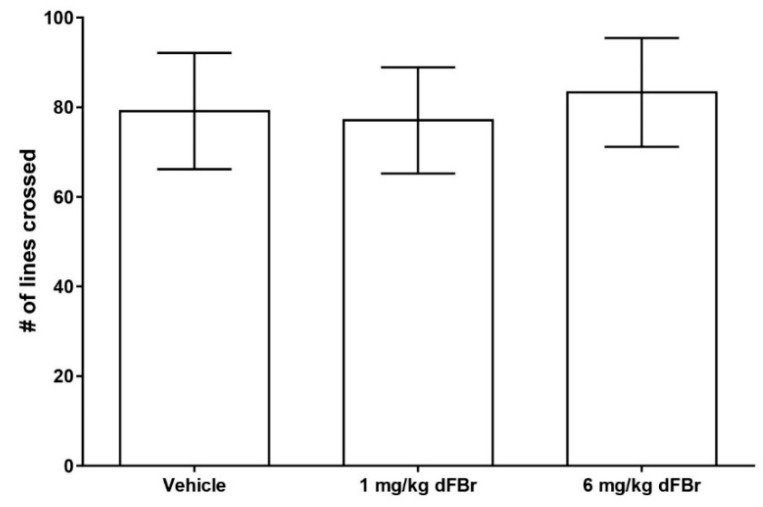
Desformylflustrabromine (dFBr) does not cause a significant change in the number of lines crossed in the open field exploration test. The number of lines crossed is expressed as mean (±S.D.). Statistical significance was considered as *p* < 0.05. Twelve mice were included in each treatment group (*n* = 12).

**Figure 5:**
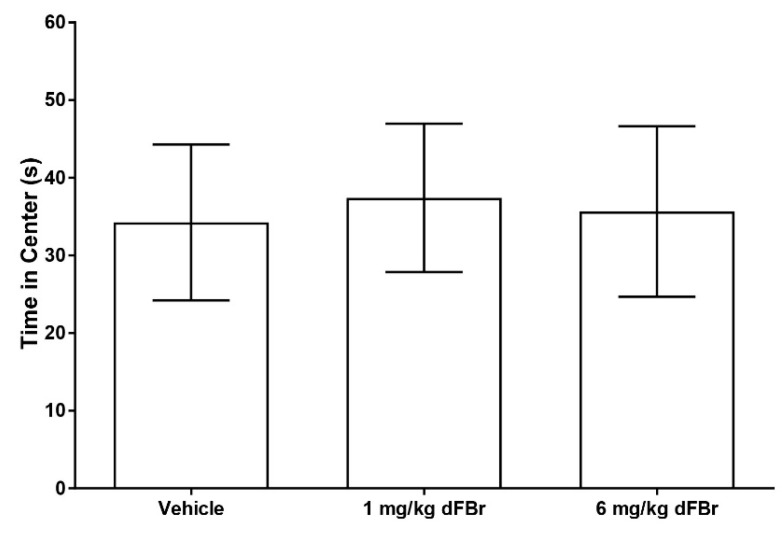
Desformylflustrabromine does not cause a significant change in the time spent in the center of the arena by CD-1 mice in the open field exploration test. The time spent in the center is expressed in seconds as mean (±S.D.). Statistical significance was considered as *p* < 0.05. Twelve mice were included in each treatment group (*n* = 12).

The open field exploration test is a method of studying exploration patterns and anxiety in rodents [17,28]. In this study, the number of lines crossed was used as a measure of exploratory behavior in CD-1 mice (Figure 4). Both 1 mg/kg and 6 mg/kg of dFBr failed to produce any significant change in the number of lines crossed by the mice (F (2.33) = 0.7993; *p* = 0.4581). The amount of time spent in the center zone by the mice was used as a measure of anxiety in the open field test. (Figure 5). Both doses of 1 mg/kg and 6 mg/kg of dFBr that were tested failed to produce any significant change in the amount of time the mice spent in the center of the open field (F (2.33) = 0.29; *p* = 0.7501). Finally, no treatment-related adverse clinical signs were observed in the mice at the doses of dFBr that were used in these behavioral tests.

## 3. Discussion

In the present study, dFBr was tested for the ability to inhibit pain induced by chemical means. Additionally, the effects of dFBr on locomotion and anxiety were studied in the open field exploration test. The time spent in the center during the open field test was used as an indicator of anxiety in this test. The results showed that dFBr had no effect on anxiety-like behavior, since there was no increase or decrease in the amount of time the mice spent in the center of the open field. This effect is similar to that observed in a previous study that reported no difference in the time spent in the center of the open field between mice treated with dFBr or a control vehicle [28]. Both acute and chronic administration of dFBr have no effect on anxiety when assessed in the open field test [28]. Taken together, the results from the open field test obtained in this study and those from the previous study clearly indicate that dFBr has no effect on anxiety.

The results from the open field exploration test indicate that acute administration of dFBr does not affect the locomotor activity in mice, since there was neither an increase nor decrease in locomotion due to the acute administration of dFBr. A previous study has assessed the effect of acute administration of dFBr on locomotion and anxiety-like behavior in the open field test [28]. The results of our study are in agreement with the observation of the previous study that acute administration of dFBr does not change locomotor activity. Nicotine administration in mice has been shown to cause an increase in locomotor activity, but such an effect is missing in nAChR β2-subunit knockout mice [25,26]. Moreover, β2-subunit-containing nAChRs are also known to be involved in mediating the addictive properties of nicotine [29]. On the one hand, the increase in locomotion by nicotine is due to its ability to activate the dopaminergic neurons within the ventral tegmental area of the nucleus accumbens [26,30,31]. On the other hand, nicotine acts on the presynaptic α4β2 receptors which cause the release of dopamine from the axonal terminals on neurons located in the ventral tegmental area that project to the nucleus accumbens, which ultimately leads to nicotine addiction [32,33,34]. Therefore, the locomotion-enhancing actions of nicotine seem to be related to the development of nicotine addiction through the release of dopamine in the nucleus accumbens. Studies with dFBr on rats have shown that this drug does not have any addictive effects, since it cannot sustain a self-administration behavior in mice [23,35]. While dFBr is a PAM, nicotine is an agonist; therefore, they have a different mechanism of pharmacological action on the α4β2 receptors [23]. Furthermore, a study previously carried out with NS-9283, another PAM for the α4β2 receptors, produced no effect on locomotor activity [19]. Taken together, our study with dFBr and the previous study with NS-9283 indicate that PAMs for α4β2 receptors have no effect on locomotion in the open field exploration test. Unlike nicotine, dFBr likely cannot induce the release of dopamine in the nucleus accumbens, which is linked not only to the development of addiction but also to the increase in locomotor activity.

The other aspect of this study was to assess if acute administration of dFBr was able to produce an analgesic effect in two chemical assays for nociception in mice. Due to the tryptamine scaffold, there is a structural similarity between serotonin and dFBr. The action of serotonin in modulating the sensation of pain is well documented and it involves multiple different serotonin receptors [36]. The results from the formalin test and writhing test clearly show that acute doses of dFBr are able to produce a significant analgesic effect in the mice. The analgesic efficacy of dFBr was comparable to that of meloxicam, which is a clinically used NSAID in humans and in veterinary care. Furthermore, both doses of dFBr (1 mg/kg and 6 mg/kg) that were tested here produced analgesia, suggesting that the effective concentration of dFBr to relieve pain is lower than the 1 mg/kg dose that was tested. The α4β2 receptors present in the brain and the spinal cord are involved in the processing of pain, like those found in the descending monoaminergic inhibitory pain pathway [9]. The presynaptic α4β2 receptors located on neurons in the midbrain periaqueductal gray (PAG) regulate the release of gamma aminobutyric acid (GABA) from the axonal terminals [37]. The midbrain periaqueductal gray is part of one of the main elements of the descending pain inhibitory pathways [37]. Similarly, the presynaptic α4β2 receptors modulate the spinal GABAergic neurons, which are involved in transmitting the sensory signal of nociception to the brain [11,38]. Moreover, the β2-subunit of nAChRs are involved in the inhibitory control of nociception in the spinal cord [39]. In addition to the modulation of GABA release, the presynaptic α4β2 receptors are involved in the release of serotonin and norepinephrine in the spinal cord, which are primarily involved in the descending inhibitory pathway that regulates nociceptive signaling in the central nervous system [40,41]. dFBr, through its allosteric action, increases the response profile of presynaptic α4β2 receptors that are activated by endogenous acetylcholine in the synapse. Such an action of dFBr can increase the synaptic tone of neurons in the descending pain inhibitory pathway that would be seen as an analgesic effect in a whole organism.

A prior study with dFBr investigated its effects on mechanical allodynia in the chronic constrictive nerve injury (CCI)-induced neuropathic pain [24]. In this study, dFBr alone did not have any effect on neuropathic pain [24]. However, when dFBr was administered with nicotine, a dose-dependent potentiation of the anti-nociception effect caused by nicotine was observed [24]. In our study, dFBr alone in acute doses was able to produce an anti-nociception effect in the two tests that represent chemically induced pain. The difference in the effects of dFBr on neuropathic pain and chemically induced pain may simply be due to the different nature of these pain models. dFBr in acute doses is able to reduce the compulsive-like behaviors of nest building and marble burying in a non-induced compulsive-like mouse model [28]. This observation indicates that dFBr is able to produce its effects in rodents by itself without the requirement of an exogenous agonist.

## 4. Methods and Materials

### 4.1. Animals

In total, 140 male CD-1 mice were obtained that were approximately 22–24 g in weight (Charles River laboratories international, Inc. Wilmington, MA, USA). The mice were allowed to acclimate to the animal facilities for at least one week prior to their use. The mice were housed individually in one room with 12 h/12 h light/dark cycle (lights on from 7:00 a.m. to 7:00 p.m.) and given food and water ad libitum. The temperature of the room was maintained at 22 ± 3 °C with humidity at 50 ± 10%. While the mice were housed, dosed, and tested in three separate adjacent rooms, the humidity and temperature in all the three rooms was similar. Procedures pertaining to the use of the CD-1 mice were reviewed and approved by the Animal Care and Use Committee (IACUC) of the University of Alaska Fairbanks (UAF).

### 4.2. Drug Treatment

The test drug dFBr was purchased from Tocris, Inc. (Bristol, UK). Meloxicam was used as a positive control in both tests for nociception. For the formalin test, 20 μL of 5% formaldehyde solution prepared in 0.9% sterile saline was injected subcutaneously into the dorsal surface of the right hind paw of each mouse. For the writhing test, 1% acetic acid solution was prepared in 0.9% sterile saline, and 0.1mL/10g body weight was administered to each mouse via intraperitoneal (i.p.) injections. Solutions of dFBr for injection were made in 0.9% sterile saline. Two solutions of 1 mg/mL and 0.1 mg/mL were prepared for the doses of 6 mg/kg and 1 mg/kg of dFBr, respectively. The dose of dFBr that was included for testing was based on the previously reported dose that was safe and effective in reducing nicotine self-administration in male Sprague–Dawley rats [35].

For meloxicam, a solution of 0.1 mg/mL was prepared by diluting a commercially (Eloxiject (meloxicam), manufacturer: Henry Schein^®^ Animal Health, Dublin, OH, USA) available solution of 5 mg/mL in 0.9% sterile saline. The dose of meloxicam administered to each mouse was 20 mg/kg. The volumes of dFBr and meloxicam solution injected were according to the weight of each individual mouse. Meloxicam and dFBr were administered via i.p. injections, 30 min before the testing began. The route of administration as well as the time between injection and behavioral test were based on mean plasma and cerebrospinal fluid concentrations that dFBr achieved upon acute administration [35]. Similarly, the dose, route of administration, and time between injection and testing for meloxicam were based on its pharmacokinetic parameters reported previously [42].

### 4.3. Formalin-Induced Pain Test

The formalin test was carried out in an observation chamber made of transparent Plexiglas material. The mice received the drug treatment (time—0 min) 30 min prior to the formaldehyde injection. In response to the noxious stimuli of formaldehyde injection, the mice demonstrated a nocifensive behavior of licking/biting of the injected paw. The formaldehyde-induced paw-licking behavior occurs in two phases [43]. The early-phase behavioral response to pain is mainly due to the activation of C-fibers by formaldehyde injection acting as a stimulus [44]. The late-phase behavioral response to pain is due to the inflammation induced by formaldehyde and to changes in the dorsal horn of the spinal cord [44]. The mice received the formaldehyde injection 30 min after drug treatment (time—30 min). Then, 1 min later, the mice were introduced in the observation chamber to quantify the early-phase response (time—31 to 36 min). Both early-phase response and late-phase response were measured for a period of 5 min. At the end of the early phase observation, the mice were returned to their home cages. The measurement of the late-phase response (time—56 to 61 min) began 25 min after the start of the early observation, with the introduction of the mice in the observation chamber for a second time. During each observation phase, the number of times a mouse licked or bit the injected paw was counted as a measure of pain.

### 4.4. Acetic Acid-Induced Writhing Response Test

The other test performed to evaluate the anti-nociceptive ability of dFBr was the writhing test. Like the formalin test, the writhing test was also carried out in an observation chamber made of transparent Plexiglas material. The mice received the drug treatment (time—0 min) 30 min prior to the i.p. injection of acetic acid. In response to the noxious stimuli of acetic acid in the peritoneum, the mice demonstrated a writhing response consisting in visceral muscle contractions seen as writhes [45]. The mice received the acetic acid injection 30 min after drug treatment (time—30 mins). Then, 5 min later, the mice were introduced in the observation chamber to quantify the writhing response. The observation period for this test was 10 min (time—35 to 45 min). During the observation period, the number of writhes induced by the acetic acid injection was counted as a measure of pain. A writhe was defined as arching of the back, pelvic rotation, and/or hind limb extension.

### 4.5. Open Field Exploration Test

The effects of dFBr on anxiety and locomotor activity were measured by the open field exploration test carried out in a cube-shaped transparent Plexiglas box, which was 57 cm-long on all sides. The base of the box was divided into nine quadrants of 19 cm^2^ each that were made by drawing two horizontal and vertical lines. The mice received the drug treatment (time—0 min) 30 min prior to the testing period. The behavior of all mice in this test was video-recorded with a digital camera (Logitech HD Pro Webcam C920) that was attached to a laptop.

At the beginning of the test period, the mice were introduced in the open arena by placing them in one corner of the box. All mice were introduced by placing them in same corner of the box to avoid variability. The mice were allowed to move freely for a period of 5 min during the test period (time—30 to 35 min). In order to measure the locomotor activity, the number of lines crossed by the mice during the 5 min test period was counted. A single line cross was counted when any two or all four paws of a mouse crossed a line. For assessing anxiety, the amount of time a mouse spent in the central quadrant of the box during the 5 min testing period was scored by reviewing the video recording of each mice during the open field test.

### 4.6. Data Analysis

Data from the behavioral tests were analyzed using ordinary one-way ANOVA and Turkey’s multiple comparison post-hoc test where all the variables obtained were compared to each other. Additionally, the two-tailed Student’s *t*-test was also used to measure differences between two variables in the behavioral tests. All statistical differences were deemed significant at the level of *p* < 0.05. Statistical analysis was carried out using GraphPad Prism 6.05 Software (San Diego, CA, USA).

## 5. Conclusions

The involvement of the α2β4 receptor in pain signaling makes them a suitable target for the development of new analgesic drugs. Many agonists and partial agonists for the α4β2 receptors have been studied in animal models of pain for their anti-nociceptive actions. This study demonstrated that PAMs for the α2β4 receptors, like dFBr, need vigorous and detailed investigation since they have clear potential to become clinically useful drugs for relieving pain.
